# Evaluation of Prescription Drug Monitoring Program Integration With Hospital Electronic Health Records by US County-Level Opioid Prescribing Rates

**DOI:** 10.1001/jamanetworkopen.2020.9085

**Published:** 2020-06-29

**Authors:** A. Jay Holmgren, Nate C. Apathy

**Affiliations:** 1 Harvard Business School, Boston, Massachusetts; 2 Richard M. Fairbanks School of Public Health, Indiana University, Indianapolis

## Abstract

This cross-sectional study assesses the level of electronic health record (EHR) and prescription drug monitoring program integration in hospitals in US counties with vs without high opioid prescribing rates.

## Introduction

Prescription drug monitoring programs (PDMPs) have become a widely embraced policy solution to the opioid epidemic in the US. PDMPs offer prescribers a comprehensive view of patients’ controlled substance prescription history and can be used to monitor and reduce inappropriate opioid prescribing.^1^ However, poor usability and lack of integration with electronic health records (EHRs) have limited their effectiveness.^2^ Furthermore, without PDMP integration in EHRs, prescribers are forced to manage multiple disconnected software systems that interrupt clinical workflow, which may exacerbate technology-driven physician burnout and result in prescribers neglecting to check the PDMP before writing opioid prescriptions.^3^ Efforts to integrate PDMPs with EHRs may be associated with reduced prescriber burden and improved PDMP effectiveness; however, the extent to which this adoption has occurred remains unknown on a national scale in the US. Extant literature has focused on usability of PDMP systems rather than adoption and integration. PDMP integration rates are particularly important among hospitals, which are major sources of ambulatory care and thus potential opioid prescribing. Furthermore, hospitals in areas with high opioid prescribing rates may gain the most from PDMP integration efforts because physicians are more likely to provide treatment to patients with current or past opioid prescriptions in these regions. In this study, we assessed the level of EHR and PDMP integration in hospitals, comparing hospitals located in US counties with vs without high opioid prescribing rates.

## Methods

We conducted a cross-sectional study using newly available national hospital data from the American Hospital Association Annual Survey and IT Supplement for 2018 (data from 2018 were collected in 2019) to evaluate the following 3 PDMP capabilities: ability to electronically prescribe controlled substances (opioid e-prescribe), ability to check the PDMP from within the EHR (PDMP query), and whether the EHR automatically integrates data from the PDMP (PDMP integration). We also assessed hospital characteristics, including size, ownership, teaching status, system membership, rurality, and region.^4^ We combined this with county-level data on opioid prescribing rates (number of opioid prescriptions dispensed per 100 residents) for 2017 to identify whether each hospital was located in a high (top-quartile) opioid prescribing county.^5^ Our analytic sample included 3512 hospitals. This study does not involve human subjects research; thus, it was deemed exempt from institutional review board review and informed consent was waived. This study followed the Strengthening the Reporting of Observational Studies in Epidemiology (STROBE) reporting guideline.

We first calculated bivariate comparisons for each of the 3 hospital PDMP capabilities, comparing hospitals located in counties with high rates of opioid prescribing with those located in other counties. We used χ^2^ tests for statistical significance. We then ran 3 multivariable logistic regression models, with 1 for each PDMP capability as a dependent variable. Our primary independent variable was location in a county with a high rate of opioid prescribing. We adjusted for hospital characteristics with robust SEs clustered at the county level.^6^ We plotted average marginal effects (AME) and 95% confidence intervals (CI) on forest plots. The analysis was conducted using Stata, version 16 (StataCorp LLC) and R, version 3.6.3 (R Project for Statistical Computing). Statistical significance was considered as *P* = .05 using 2-sided tests.

## Results

The sample included 3512 hospitals, of which 639 (18.2%) were located in counties with high rates of opioid prescribing and 2873 (81.8%) in other counties. Most hospitals were able to e-prescribe controlled substances, although hospitals in the top quartile of opioid prescribing counties were less likely to be able to do so (366 [57.3%] vs 1874 [65.2%] of other hospitals; *P* < .001). Electronic health record–based PDMP queries were less common overall and available at 141 hospitals (22.1%) located in counties with high rates of opioid prescribing compared with 871 other hospitals (30.3%) (*P* < .001). Only 69 hospitals (10.8%) in counties with high rates of opioid prescribing reported PDMP integration compared with 434 other hospitals (15.1%) (*P* = .01) (Table).

**Table.** Hospital Characteristics by Overall Sample and Location in Counties With High Rates of Opioid Prescribing

Characteristic	No. (%)			*P* value^a^
	Overall sample (N = 3512)	High opioid prescribing county (n = 639)	Other county (n = 2873)	
PDMP capabilities				
Hospital enabled for electronic prescribing of controlled substances	2240 (63.8)	366 (57.3)	1874 (65.2)	<.001
Prescribers can check state PDMP via their EHR	1012 (28.8)	141 (22.1)	871 (30.3)	<.001
Hospital EHR automatically integrates data from state PDMP	503 (14.3)	69 (10.8)	434 (15.1)	.01
Hospital size				
Small, <100 beds	1745 (49.7)	362 (56.7)	1383 (48.1)	<.001
Medium, 100-399 beds	1370 (39.0)	236 (36.9)	1134 (39.5)	
Large, ≥400 beds	397 (11.3)	41 (6.4)	356 (12.4)	
Teaching status				
Teaching hospitals	1379 (39.3)	172 (26.9)	1207 (42.0)	<.001
Nonteaching hospitals	2133 (60.7)	467 (73.1)	1666 (58.0)	
System membership				
Hospitals part of a health care system	2405 (68.5)	435 (68.1)	1970 (68.6)	<.001
Nonsystem hospitals	1107 (31.5)	204 (31.9)	903 (31.4)	
Location				
Rural	1114 (31.7)	308 (48.2)	806 (28.1)	<.001
Urban	2398 (68.3)	331 (51.8)	2067 (71.9)	
Region				
West	560 (15.9)	42 (6.6)	518 (18.0)	<.001
Midwest	1113 (31.7)	150 (23.5)	963 (33.5)	
South	1305 (37.2)	441 (69.0)	864 (30.1)	
Northeast	512 (14.6)	6 (0.9)	506 (17.6)	
Ownership				
Private, nonprofit	2077 (59.1)	329 (51.5)	1748 (60.8)	<.001
Private, for-profit	691 (19.7)	156 (24.4)	535 (18.6)	
Public, nonfederal	685 (19.5)	147 (23.0)	538 (18.7)	
Federal	59 (1.7)	7 (1.1)	52 (1.8)	

Abbreviations: EHR, electronic health record; PDMP, prescription drug monitoring program.

^a^ *P* values derived from Rao-Scott omnibus χ^2^ tests.

Multivariable models had similar results (Figure). Hospitals located in counties with high rate of opioid prescribing were less likely to report e-prescribing of controlled substances (AME, −0.05; 95% CI, −0.09 to 0.00; *P* = .04), EHR-based PDMP queries (AME, −0.07; 95% CI −0.12 to −0.02; *P* < .01), and PDMP integration (AME, −0.05; 95% CI, −0.09 to −0.01; *P* = .02).

**Figure.**
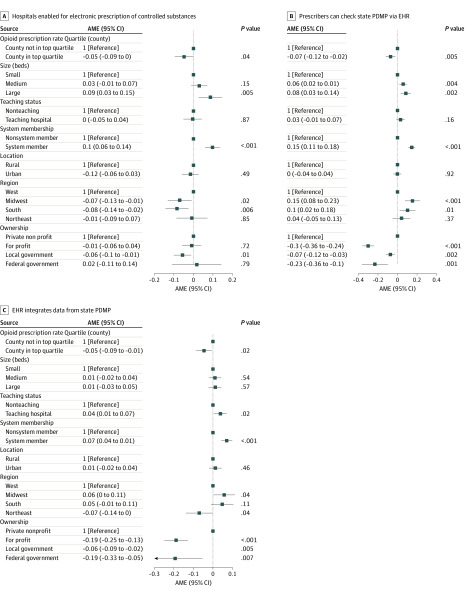
Multivariable Logistic Regression Models Forest plot shows average marginal effects (AMEs) and 95% CIs of 3 multivariable logistic regression models. All models included robust SEs clustered at the county level. Squares indicate estimates, and horizontal lines indicates 95% CIs. EHR indicates emergency health record; PDMP, prescription drug monitoring program.

## Discussion

In this cross-sectional study, although most hospitals were able e-prescribe controlled substances, few hospitals enabled prescribers to query the PDMP from within the EHR or integrate PDMP data into their EHR. Hospitals located in areas with high rates of opioid prescribing were less likely to have these functionalities, which may limit the effectiveness of PDMPs in these high-needs areas.

This study has limitations. The analysis could not identify the causal mechanism for differences in PDMP integration, our data did not include hospital-level opioid prescribing, and we lacked data on nonhospital ambulatory physicians.

Physicians and policy makers are actively considering new strategies to manage the opioid epidemic. Focusing efforts on hospitals in areas with high rates of opioid prescribing to build PDMP integration into their EHR may be associated with improved PDMP effectiveness and reduced prescriber administrative burden.
